# The burden of mild cognitive impairment attributable to physical inactivity in Colombia

**DOI:** 10.1186/s11556-022-00307-y

**Published:** 2022-11-09

**Authors:** Gary O’Donovan, I-Min Lee, Mark Hamer, Patricia García-Garro, Claudia Duran-Aniotz, Agustín Ibáñez, Olga L. Sarmiento, Philipp Hessel

**Affiliations:** 1 Latin American Brain Health Institute (BrainLat), Universidad Adolfo Ibáñez, Santiago, Chile; 2 Facultad de Medicina, Universidad de los Andes, Bogotá, Colombia; 3 Division of Preventive Medicine, Brigham and Women’s Hospital, Harvard Medical School, Boston, MA USA; 4 Department of Epidemiology, Harvard TH Chan Medical School of Public Health, Boston, MA USA; 5 Division of Surgery & Interventional Science, Faculty of Medical Sciences, Institute Sport Exercise Health, University College London, London, UK; 6 Facultad de Educación a Distancia y Virtual, Institución Universitaria Antonio José Camacho, Cali, Colombia; 7 Center for Social and Cognitive Neuroscience, School of Psychology, Universidad Adolfo Ibáñez, Santiago, Chile; 8 Global Brain Health Institute, University of California, San Francisco, USA; 9 Trinity College Dublin, Dublin, Ireland; 10 Centro de Neurociencias Cognitivas, Universidad de San Andrés, Argentina and National Scientific and Technical Research Council (CONICET), Buenos AiresBuenos Aires, Argentina; 11 Trinity College Institute of Neuroscience (TCIN), Trinity College Dublin, Dublin, Ireland; 12 Swiss Tropical and Pubic Health Institute, Basel, Switzerland

**Keywords:** Physical activity, Exercise, Sports, Cognition, Primary prevention

## Abstract

**Background:**

Mild cognitive impairment often precedes dementia. The purpose of this analysis was to estimate the population attributable fraction for physical activity in Colombia, which is the reduction in cases that would occur if all participants were physically active.

**Methods:**

The sample included 20,174 men and women aged 70.04 ± 7.68 years (mean ± SD) from the National Survey of Health, Wellbeing and Ageing. Trained interviewers administered a shorter version of the mini-mental state examination and mild cognitive impairment was defined as a score of 12 or less out of 19. Logistic regression models were fitted and population attributable fractions for physical activity were calculated. All analyses were adjusted for age, sex, height, education, income, civil status, smoking, and alcohol drinking.

**Results:**

The prevalence of physical activity was approximately 50% when defined as walking between 9 and 20 blocks at least three times per week. Theoretically, 19% of cases of mild cognitive impairment would be eliminated if all adults were to walk (95% confidence interval: 16%, 22%). The prevalence was approximately 20% when defined as taking part in vigorous sport or exercise at least three times per week. Theoretically, 23% of cases of mild cognitive impairment would be eliminated if all adults were to take part in vigorous sport or exercise (16%, 30%). Similar results were observed after removing those who reported mental health problems.

**Conclusion:**

Physical activity, whether walking or vigorous sport and exercise, has the potential to substantially reduce the burden of mild cognitive impairment in Colombia.

## Background

The number of people living with dementia is predicted to increase from around 57 million cases globally in 2019 to around 153 million cases in 2050 [1]. The proportion of people living with dementia is predicted to increase by around 75% in the UK and other countries in western Europe and by around 200% in Colombia and other countries in Latin America [1]. It is important to identify modifiable risk factors because a five-year delay in onset might halve the prevalence of dementia [2]. However, nearly all the evidence about potentially modifiable risk factors for cognitive impairment comes from studies in high-income countries [3]. Indeed, the authors of the 2020 Lancet Commission on Dementia Prevention, Intervention, and Care concluded that there was an urgent need for more evidence from Latin America [3]. Much of the work in identifying modifiable risk factors for dementia has focussed on estimating the population attributable fraction, which is the proportional reduction in cases that would occur if a particular risk factor were eliminated [3, 4]. For example, it has been estimated that approximately 8% of cases of dementia would be eliminated if all adults were physically active [5]. It is plausible that physical activity improves brain health and several potential mechanisms have been identified [6–11]. For example, exercise may increase brain-derived neurotrophic factor concentrations and brain plasticity [6]. Physical activity is also associated with greater brain volume, greater executive function, and greater memory [7]. It may be particularly important to identify modifiable risk factors for mild cognitive impairment because mild cognitive impairment often precedes dementia [3, 4]. However, to the best of our knowledge, there are no estimates of the proportional reduction in cases of mild cognitive impairment that would occur if all adults in Colombia were physically active. It is important to gather evidence from each country in Latin America because the region is so diverse and so difficult to understand [12]. Colombia may be particularly difficult to understand because the country has suffered 50 years of civil war [13]. Therefore, the aim of the present analysis was to calculate the burden of mild cognitive impairment that is attributable to physical inactivity in Colombia. We used data from the National Survey of Health, Wellbeing and Ageing in Colombia (SABE Colombia, according to its initials in Spanish), which is the largest nationally representative survey of older adults in Colombia [14].

## Methods

### Participants

The National Survey of Health, Wellbeing and Ageing in Colombia is described in detail elsewhere [14]. Briefly, the target population was all adults aged 60 years or older living in households. Participants were selected using a multistage area probability sampling design and there were four selection stages: municipalities, blocks, housing units, and households. The response rate was around 62% in urban areas, around 77% in rural areas, and around 70% overall [14]. Data were collected across all departments (that is, states) and the final sample was deemed to be representative of the population of older adults living in households in Colombia [14]. Trained interviewers conducted face-to-face interviews in the participant’s home between April and September 2015 [14]. Volunteers completed the shorter version of the Folstein Mini-Mental State Examination (MMSE) described below and were invited to participate in the interview if they had a score of 13 or more [14]. Otherwise, a friend or family member was invited to complete the interview on behalf of the participant. A friend or family member completed the interview in 17.5% of cases [14]. Institutional review boards of *Universidad de Caldas* and *Universidad del Valle* approved the study and all participants gave written informed consent.

### Outcome

The outcome variable was mild cognitive impairment. The versions of the MMSE used in Latin America and the Caribbean are shorter than the original version in an attempt to reduce the low literacy bias [15]. The shorter version of the MMSE used in SABE Colombia has six questions and participants were asked: to state the date and the day of week (4 points); to repeat and remember three words (3 points); to state in reverse order the numbers 1, 3, 5, 7, 9 (5 points); to take a piece of paper in their right hand, fold it in half using both hands, and put it on their lap (3 points); to reiterate the three words given earlier (3 points); and, to copy a drawing of two overlapping circles (1 point). A score of 12 or less out of 19 was used to screen for mild cognitive impairment in SABE Colombia [14]. The shorter version of the MMSE used in SABE Colombia has been validated in a study of 1,301 adults aged 60 years or older living in households in Chile [16]. The prevalence of mild cognitive impairment was 10.7% using the threshold of 12 or less out of 19 in the shorter version of the MMSE and 8.1% using the threshold of 6 or more out of 33 in the criterion measure [16], which was the Short Portable Mental Status Questionnaire [17].

### Exposure

The exposure was physical activity. Participants were asked two questions about physical activity. First, participants were asked if they took part in vigorous sport or exercise at least three times per week: “Do you participate at least three times per week in any sporting activity or do you do exercises like swimming, jogging, playing tennis, riding a bicycle, aerobics, gymnastics classes, or other activities that cause you to sweat or that leave you out of breath?” Then, participants were asked if they walked between 9 and 20 blocks at least three times per week without stopping. Answers were “yes” or “no”. Single-item physical activity assessment tools like the one used in the present study have been validated against multiple-item physical activity assessment tools [18] and against cardiorespiratory fitness [19].

### Covariates

The analyses were adjusted for a range of covariates that may be associated with cognitive decline, including age, sex, height, education, income, civil status, cigarette smoking, and alcohol drinking [20–22]. Participants were asked about the highest level of education they had achieved, and three groups were created: no education; some primary education; and, some secondary education or more. Participants were also asked about their current individual income according to multiples of the minimum wage. Participants were asked about their current civil status, and two groups were created: not married or with partner; and, married or with partner. Participants were asked about cigarette smoking, and three groups were created: never, former smoker, and current smoker. Participants were also asked about alcohol drinking in the last month, and two groups were created: non-drinker; and, drinker. The trained interviewers also measured height.

The analyses were further adjusted for potential risk factors for dementia, including body mass index, heart disease, and cholesterol concentrations [20]. Trained interviewers measured weight and height, and body mass index was expressed as weight in kilogrammes divided by height in metres squared. Body mass index values greater than 75 were deemed to be dubious and were not included in the present analysis. Participants were asked if a doctor had ever told them that they had had a heart attack or other heart problem. A sub-sample of more than 4,000 participants in SABE Colombia gave a blood sample after an overnight fast. Around 1.5% of blood samples were not included in the present analyses because total cholesterol concentrations or high-density lipoprotein cholesterol concentrations were outside the reference ranges for older adults [23].

Finally, we considered the problem of reverse causation. There is a risk of reverse causation because a decrease in physical activity may occur prior to a diagnosis of cognitive decline or dementia as part of the disease process [24, 25]. Participants were asked if a doctor or a nurse had ever told them that they had had a mental health problem. Participants who said yes were removed to consider the problem of reverse causation.

### Statistical analyses

All analyses were performed using Stata MP version 15.1. for Mac (StataCorp, Texas, USA). Logistic regression was used to investigate associations between physical activity and mild cognitive impairment. Separate models were created for the exposure variable ‘walking between 9 and 20 blocks at least three times per week’ (yes or no) and the exposure variable ‘taking part in vigorous sport or exercise at least three times per week’ (yes or no). Logistic regression models were adjusted for age, sex, height, education, current individual income, civil status, cigarette smoking, and alcohol drinking. Age was modelled as a continuous variable. All other covariates were modelled as categorical variables. Odds ratios and 95% confidence intervals were calculated for all participants and were also stratified by sex because the decline in cognitive function may be greater in older men [26]. The likelihood-ratio chi-squared test for each model was highly significant (each *P* < 0.001). The *punaf* command in Stata is used to estimate the population attributable fraction after fitting a logistic regression model in a cross-sectional study [27]. In the present analysis, the *punaf* command was used to estimate the proportion of mild cognitive impairment cases that were attributable to physical inactivity. Physical activity was defined as either walking between 9 and 20 blocks at least three times per week or taking part in vigorous sport or exercise at least three times per week. We estimated the proportional reduction in mild cognitive impairment cases that would occur if all participants were physically active and all other variables in the model stayed the same. In sensitivity analyses, the original models were further adjusted for body mass index, heart disease, and cholesterol concentrations; body mass index and cholesterol concentrations were modelled as continuous variables and heart disease was modelled as a categorical variable. Finally, participants who reported mental health problems were removed to consider the problem of reverse causation.

## Results

Table 1 shows participants’ characteristics. There were some notable differences between those who were and were not included in the present analysis. For example, those who were included in the present analysis were more likely to be physically active than those who were not; and, those who were included were more likely to have received some education and were less likely to have mild cognitive impairment than those who were not. The analytic sample included more than 20,000 men and women of around 70 years of age (range: 60 to 108 years). Most participants earned less than the minimum wage, around half were married or with a partner, around half had never smoked, and few were alcohol drinkers. There were few missing values other than height, which was not measured in more than 3,000 participants.

**Table 1:** Characteristics of survey participants who were and were not included in the present analysis

	Included	Not included
Physically active, walking between 9 and 20 blocks at least three times per week		
No, n (%)	9,916 (49.15)	2,476 (70.34)
Yes, n (%)	10,255 (50.83)	1,021 (29.01)
Missing, n (%)	3 (0.01)	23 (0.65)
Total, n (%)	20,174 (100)	3,520 (100)
Physically active, taking part in vigorous sport or exercise at least three times a week		
No, n (%)	16,210 (80.35)	3,151 (89.52)
Yes, n (%)	3,963 (19.64)	365 (10.37)
Missing, n (%)	1 (0.01)	4 (0.11)
Total, n (%)	20,174 (100)	3,520 (100)
MMSE score, mean ± SD (n)	15.35 ± 3.53 (20,174)	12.60 ± 4.98 (3,520)
Mild cognitive impairment		
No, n (%)	16,914 (83.84)	2,090 (59.38)
Yes, n (%)	3,260 (16.16)	1,430 (40.62)
Missing, n (%)	0 (0)	0 (0)
Total, n (%)	20,174 (100)	3,520 (100)
Age in years, mean ± SD (n)	70.04 ± 7.68 (20,174)	75.31 ± 9.54 (3,520)
Sex		
Male, n (%)	8,913 (44.18)	1,199 (34.06)
Female, n (%)	11,261 (55.82)	2,321 (65.94)
Missing, n (%)	0 (0)	0 (0)
Total, n (%)	20,174 (100)	3,520 (100)
Height in cm, mean ± SD (n)	156 ± 9 (20,174)	155 ± 9 (411)
Current income		
None, n (%)	3,093 (15.33)	643 (18.27)
Less than minimum wage, n (%)	11,620 (57.60)	1,848 (52.50)
Minimum wage, n (%)	2,788 (13.82)	380 (10.80)
1 to 2 times minimum wage, n (%)	1,703 (8.44)	203 (5.77)
2 to 3 times minimum wage, n (%)	519 (2.57)	51 (1.45)
3 to 4 times minimum wage, n (%)	249 (1.23)	13 (0.37)
More than 4 times minimum wage, n (%)	202 (1.00)	27 (0.77)
Missing or did not answer, n (%)	0 (0)	355 (10.09)
Total, n (%)	20,174 (100)	3,520 (100)
Civil status		
Not married or with partner, n (%)	9,092 (45.07)	2,035 (57.81)
Married or with partner, n (%)	11,082 (54.93)	1,475 (41.90)
Missing or did not answer, n (%)	0 (0)	10 (0.28)
Total, n (%)	20,174 (100)	3,520 (100)
Education		
None, n (%)	4,218 (20.91)	1,011 (28.72)
Some primary, n (%)	11,593 (57.47)	1,869 (53.10)
Some secondary or more, n (%)	4,363 (21.63)	547 (15.54)
Missing, n (%)	0 (0)	93 (2.64)
Total, n (%)	20,174 (100.00)	3,520 (100.00)
Cigarette smoking		
Never smoked, n (%)	9,563 (47.40)	1,837 (52.19)
Former smoker, n (%)	8,390 (41.59)	1,373 (39.01)
Current smoker, n (%)	2,221 (11.01)	302 (8.58)
Missing or did not answer, n (%)	0 (0)	8 (0.23)
Total, n (%)	20,174 (100)	3,520 (100)
Alcohol drinking		
Non-drinker, n (%)	17,604 (87.26)	3,277 (93.10)
Drinker, n (%)	2,570 (12.74)	226 (6.42)
Missing or did not answer, n (%)	0 (0)	17 (0.48)
Total, n (%)	20,174 (100)	3,520 (100)

Data are from the National Survey of Health, Wellbeing and Ageing in Colombia. Participants were community-dwelling adults aged 60 years or older. MMSE is shorter mini-mental state examination. Mild cognitive impairment was defined as a score of 12 or less out of 19 on the MMSE

The prevalence of physical activity was around 50% when defined as walking between 9 and 20 blocks at least three times per week (Table 1). The fully adjusted odds ratio for mild cognitive impairment was 0.60 in those who reported walking between 9 and 20 blocks at least three times per week compared with those who did not (95% confidence interval: 0.55, 0.65). Theoretically, 19% of cases of mild cognitive impairment would be eliminated if all adults were to walk between 9 and 20 blocks at least three times per week (95% confidence interval: 16%, 22%). The prevalence of physical activity was around 20% when defined as taking part in vigorous sport or exercise at least three times per week (Table 1). The fully adjusted odds ratio for mild cognitive impairment was 0.65 in those who reported taking part in vigorous sport or exercise at least three times per week compared with those who did not (95% confidence interval: 0.57, 0.74). Theoretically, 23% of cases of mild cognitive impairment would be eliminated if all adults were to take part in vigorous sport or exercise at least three times per week (95% confidence interval: 16%, 30%).

Table 2 shows odds ratios and population attributable fractions stratified by gender. Population attributable fractions were higher in males than females when physical activity was defined as walking between 9 and 20 blocks at least three times per week, but the 95% confidence interval for the differences between men and women includes the null value of a difference of zero (-1, 11). Population attributable fractions were also higher in males than females when physical activity was defined as taking part in sport or exercise at least three times per week, but the 95% confidence interval for the differences between men and women also includes the null value of a difference of zero (-8, 18). Table 3 shows the sensitivity analyses. Population attributable fractions for physical activity were similar after further adjustment for body mass index (more than 20,000 observations for walking and for sport or exercise). Population attributable fractions were also similar after further adjustment for a diagnosis of heart disease (more than 20,000 observations for walking and for sport or exercise). The effect of cholesterol was unclear because the sample sizes were small and the confidence intervals were wide (less than 3,500 observations for walking and for sport or exercise).

**Table 2:** Odds ratios for mild cognitive impairment and population attributable fractions for physical activity stratified by gender

	Male	Female
Odds ratios for mild cognitive impairment (95% confidence interval)		
Physically active, walking between 9 and 20 blocks at least three times per week	0.51 (0.45, 0.58)	0.69 (0.61, 0.78)
Physically active, taking part in vigorous sport or exercise at least three times a week	0.61 (0.51, 0.74)	0.68 (0.57, 0.82)
Population attributable fractions for physical activity (95% confidence interval)		
Physically active, walking between 9 and 20 blocks at least three times per week	20% (16%, 24%)	15% (10%, 20%)
Physically active, taking part in vigorous sport or exercise at least three times a week	26% (16%, 35%)	21% (11%, 30%)

The odds ratios for mild cognitive impairment and the population attributable fractions for physical activity were estimated while adjusting for age, height, education, current individual income, civil status, cigarette smoking, and alcohol drinking

**Table 3:** Population attributable fraction of mild cognitive impairment due to physical activity, with further adjustment for body mass index, heart disease, and cholesterol concentrations

	Population attributable fraction (95% confidence interval)
Further adjustment for body mass index^1^	
Physically active, walking between 9 and 20 blocks at least three times per week	19% (16%, 22%)
Physically active, taking part in vigorous sport or exercise at least three times a week	23% (16%, 30%)
Further adjustment for heart disease^2^	
Physically active, walking between 9 and 20 blocks at least three times per week	19% (16%, 22%)
Physically active, taking part in vigorous sport or exercise at least three times a week	23% (16%, 29%)
Further adjustment for cholesterol^3^	
Physically active, walking between 9 and 20 blocks at least three times per week	26% (16%, 34%)
Physically active, taking part in vigorous sport or exercise at least three times a week	8% (-11%, 24%)

^1^Models adjusted for age, sex, height, education, current individual income, civil status, cigarette smoking, alcohol drinking, and body mass index (*n* = 20,093 for walking and 20,107 for sport or exercise). Body mass index values greater than 75 were deemed to be dubious and were not included

^2^Models adjusted for age, sex, height, education, current individual income, civil status, cigarette smoking, alcohol drinking, and a diagnosis of heart disease (*n* = 20,152 for walking and 20,166 for sport or exercise). More than 3,000 participants in SABE Colombia reported a diagnosis of heart disease

^3^Models adjusted for age, sex, height, education, current individual income, civil status, cigarette smoking, alcohol drinking, total cholesterol, and high-density lipoprotein cholesterol (*n* = 3,432 for walking and 3,435 for sport or exercise). More than 4,000 participants in SABE Colombia gave a blood sample after an overnight fast. Total cholesterol concentrations greater than 300 mg/dL and high-density lipoprotein cholesterol concentrations greater than 80 mg/dL were deemed to be dubious and were not included

More than 2,000 participants in SABE Colombia reported a mental health problem and were removed from the analytic sample to consider the problem of reverse causation. Similar results were observed after removing those who reported mental health problems, as follows. The fully adjusted odds ratio for mild cognitive impairment was 0.62 in those who reported walking between 9 and 20 blocks at least three times per week compared with those who did not (95% confidence interval: 0.56, 0.68). The population attributable fraction for physical activity was 18% (14%, 21%). The fully adjusted odds ratio for mild cognitive impairment was 0.65 in those who reported taking part in vigorous sport or exercise at least three times per week compared with those who did not (95% confidence interval: 0.56, 0.75). The population attributable fraction for physical activity was 23% (16%, 30%).

## Discussion

The aim of the present analysis was to calculate the burden of mild cognitive impairment that is attributable to physical inactivity in adults aged 60 years or older living in households in Colombia. We estimated the reduction in mild cognitive impairment cases that would occur if all older adults were to become physically active and other risk factors for cognitive decline were to stay the same. The main findings were that around 19% of cases of mild cognitive impairment would be eliminated if all adults were to walk at least three times per week and that around 23% of cases would be eliminated if all adults were to take part in vigorous sport or exercise at least three times per week. It is plausible that physical activity improves brain health and several potential mechanisms are mentioned below.

### What this study adds

Mild cognitive impairment often precedes dementia and it is important to identify modifiable risk factors for mild cognitive impairment [3, 4]. However, to the best of our knowledge, there are no estimates of population attributable fractions for physical activity in Colombia. Katzmarzyk and colleagues calculated that the burden of dementia attributable to physical inactivity was 10% in high-income Western countries (95% confidence interval: 3.3%, 19.1%) and 11.1% in Latin America and the Caribbean (95% confidence interval: 3.6%, 20.4%) [5]. They also calculated that the burden of dementia attributable to physical inactivity was 12.3% in Colombia (95% confidence interval: 3.8%, 23%) [5]. Katzmarzyk and colleagues used data from three relatively small studies in Colombia to estimate physical activity prevalence and they used data from outside Colombia to estimate relative risks [5, 28]. In the present analysis, we used data from more than 20,000 participants in a nationally representative study to estimate physical activity prevalence in Colombia and to calculate odds ratios. The present study suggests that physical activity is a modifiable risk factor for mild cognitive impairment in Colombia.

### Biological plausibility

The effect of exercise on cognition is usually determined in tests of cognitive function, such as tests of memory, tests of attention, and tests of executive function [29]. Experimental evidence suggests that physical exercise improves cognitive function in healthy adults of all ages [29] and in people with brain disorders [30]. Indeed, it is plausible that exercise improves brain health and several potential mechanisms have been identified, including mechanisms related to improvements in brain metabolism [6], brain structure [7], brain connectivity [8], brain vascular function [9], and brain plasticity [10]. The gut microbiome has a profound influence on cognitive function and there is also evidence of a combined effect of diet and exercise on the gut microbiome [11]. The various beneficial effects of exercise on the brain may be accumulated over several stages of life, making the brain more resilient to cognitive impairment and dementia [31–33]. The effect of exercise intensity on cognitive function is unclear because of the different methods used to prescribe exercise intensity in randomised, controlled trials [29]. Nonetheless, it is plausible that there is a dose–response relationship between exercise intensity and brain health [10]. For example, blood lactate concentrations rise with exercise intensity and lactate may be a key signalling molecule that mediates the beneficial effects of exercise on brain plasticity [34]. Experimental evidence also suggests that open-skill sports like basketball and tennis improve cognitive function greater than closed-skill exercises like swimming and walking [29]. The environment is constantly changing in open-skill sports and it is plausible that open-skill sports provides a greater stimulus to brain health than closed-skill exercises in which the environment is relatively consistent [35]. For example, brain-derived neurotrophic factor promotes brain plasticity and badminton may increase serum concentrations more than stationary cycling [36]. Physical activity was associated with reduced burden of mild cognitive impairment in the present study, whether open-skill or closed-skill. Motor training (i.e., balance, coordination, and flexibility) was not considered in the present analysis, but it has been suggested that aerobic training and motor training affect neuroplasticity and cognition in different ways [37]. First, it is suggested that aerobic training affects cognition via improvements in cardiorespiratory fitness, whereas motor training affects cognition directly [37]. Second, it is suggested that aerobic training affects neuroplasticity and cognition in a global manner, while motor training is task-specific in increasing neuroplasticity and affecting cognition [37]. There is some evidence that the effects of exercise training on cognitive function are greater in men than in women; however, it is not clear whether such differences are due to biology or behaviour [29]. The decline in cognitive function may be greater in men [26], and older men may have more to gain from exercise training than older women [29]. Population attributable fractions for physical activity were higher in older men than older women in the present study, but the 95% confidence intervals for the differences between men and women contained zero. Therefore, we cannot reject the null hypothesis of zero difference between men and women. While a physically active lifestyle may have beneficial effects on cognitive function, an inactive lifestyle may have detrimental effects. For example, physical inactivity is associated with an imbalance in the secretion of myokines and memory impairment [38]. Physical inactivity is also associated with vascular dysfunction and cognitive decline and dementia [38].

### Implications for policy and practice

It has recently been estimated that the cost of dementia in Colombia is around US$ 1158 million per year [39]. If it is assumed that mild cognitive impairment leads to dementia and that the population attributable fraction for physical activity is around 20%, as suggested in the present study, then around US$ 232 million per year would be saved if all adults were physically active (20% of 1158 is 232). These data suggest that there should be more emphasis on physical activity in subsequent iterations of the mental health act of Colombia [40]. Indeed, the COVID-19 pandemic has shown that many healthcare systems in Latin America cannot cope with the challenges of treating ageing populations [12, 41]. It is noteworthy that some cities in Colombia have physical activity policies and practices that work [28, 42]. In Bogotá, more than 120 kms of roads are closed to motor vehicles every Sunday and public holiday and the streets fill with more than one million walkers, runners, and cyclists [28]. In Medellín, up to 850,000 of 2.5 million residents use the city’s sport and exercise facilities every month free of charge [42]. The Colombian Ministry of Sport also provides free exercise classes to older adults in public spaces in various towns and cities throughout the country via its healthy lifestyles programmes [43].

### Strengths and limitations

The National Survey of Health, Wellbeing and Ageing in Colombia is the largest nationally representative study of older adults in Colombia. The present analyses were adjusted for a range of covariates and the relatively narrow confidence intervals suggest that valid inferences can be drawn about the burden of mild cognitive impairment that is attributable to physical inactivity in Colombia. The main limitation is the cross-sectional design; however, we did consider the problem of reverse causation and similar results were observed after removing those who reported mental health problems. Nonetheless, prospective cohort studies with long follow-up times are needed to confirm the associations between physical activity and cognitive impairment observed in the present study [25]. Evidence from high-income countries shows that physical activity is associated with reduced risk of dementia even in studies with more than 20 years of follow-up, which suggests that the association is not simply due to reverse causation [44]. Some variables were self-reported and are subject to biases. Questionnaires were used to assess physical activity, but it is preferable that questionnaires and accelerometers be used to assess physical activity in surveillance studies because each method has advantages and disadvantages [45–47]. Participants were asked about physical activity frequency and intensity, but not duration. There is a risk of misclassification bias with physical activity questionnaires, but single-item physical activity assessment tools like the one used in the present study have been shown to be valid and reliable [18]. Single-item physical activity assessment tools have also been shown to predict mortality in large samples like the present sample [48]. A systematic review found that 31 different measures of cognition were used across 117 trials and a panel of experts concluded that the MMSE and the Alzheimer’s Disease Assessment Scale-Cognitive Subscale were the best available measures of cognition in trials [49]. The shorter version of the MMSE used in SABE Colombia is a valid screening tool [16], but it is not a clinical diagnosis of cognitive impairment. Analyses were adjusted for several potential risk factors for cognitive decline, but were not adjusted for diet [20]. However, analyses were adjusted for height, which is a measure of net nutrition in early life and may be associated with cognitive function in later life [22]. The analytic sample included more than 20,000 older adults, but an even larger study may be necessary to determine whether population attributable fractions for physical activity are different in men and women. The main findings were similar after further adjustment for body mass index and heart disease; however, the effect of cholesterol was unclear because the sample sizes were small. The SABE Colombia participants who were included in the present analysis may have been healthier and more educated than those who were not included and population attributable fractions may have been underestimated.

## Conclusion

Mild cognitive impairment often precedes dementia and it is important to identify modifiable risk factors for mild cognitive impairment. The National Survey of Health, Wellbeing and Ageing in Colombia is a large, nationally representative study that includes measures of physical activity and mild cognitive impairment. The present analysis included more than 20,000 participants in SABE Colombia and the results suggest that physical activity is a modifiable risk factor for mild cognitive impairment in Colombia. Physical activity has the potential to substantially reduce the burden of cognitive decline in Colombia, whether by walking or taking part in vigorous sport and exercise. More should be done to facilitate physical activity in Colombia because the proportion of people living with dementia is predicted to increase dramatically by 2050 [1].

## Data Availability

Requests to access the data should be directed to the Colombian Ministry of Health (repositorio@minsalud.gov.co). The original name of the study is in Spanish: Salud, Bienestar & Envejecimiento (SABE Colombia). The analysis plan is available from the corresponding author.

## Abbreviations

MMSE: Is mini-mental state examination
SABE: Colombia is the National Survey of Health, Wellbeing and Ageing in Colombia
